# A Novel Surgical Hybrid Approach to Neoplastic Lesions in the Distal Part of the Urethra: A Pilot Series of Clinical Cases

**DOI:** 10.3390/ani13061074

**Published:** 2023-03-16

**Authors:** Przemysław Prządka, Agnieszka Antończyk, Bartłomiej Liszka, Wojciech Borawski, Stanisław Dzimira, Zdzisław Kiełbowicz, Ludwika Gąsior

**Affiliations:** 1 Department and Clinic of Surgery, Faculty of Veterinary Medicine, Wroclaw University of Environmental and Life Sciences, Pl. Grunwaldzki 51, 50-366 Wroclaw, Poland; 2 Department of Pathology, Division of Pathomorphology and Veterinary Forensics, Faculty of Veterinary Medicine, Wroclaw University of Environmental and Life Sciences, C.K. Norwida 31, 50-375 Wroclaw, Poland

**Keywords:** urology, laparoscopy, surgery, cancer, endoscopy, animals

## Abstract

**Simple Summary:**

Distal urethral neoplasms in female dogs are difficult to treat, due to their anatomical location and often late diagnosis. Transitional cell carcinomas are among the most recognized tumors. Available surgical techniques are difficult to perform when the tumors occupy most of the urethral length, because of the lack of adequate tissue margin and high tissue tension. Another approach is purely palliative treatment. In this article the authors propose a solution to this surgical problem with the aforementioned tumors, which is a novel hybrid technique, developed on cadaver female dogs, involving the combination of laparoscopy and open surgery. In this technique, the changed urethra is removed along with the female genitalia, with gives the opportunity to radically excise the tumor and to perform the prepubic urethrostomy. This paper presents the clinical outcomes after the treatment of urethral tumors using the hybrid technique in three female dogs. All procedures were successful, with no major complications during surgery, and the average survival time was 9 months.

**Abstract:**

All the surgical approaches described to date for the removal of distal urethral tumors have some technical difficulties that make these tumors difficult to treat. The article presents for the first time the treatment results of three female dogs, diagnosed with transitional cell carcinomas of the distal urethra, operated with a newly developed hybrid surgical method—a combination of laparoscopy and open surgery. This technique uses vulvovaginectomy, combined with resection of the distal urethra and prepubic urethrostomy. All of the procedures were possible to perform, without the need to carry out a laparotomy conversion. Histopathology revealed transitional cell carcinoma in all cases, with a margin of healthy tissues maintained in two out of three cases, which meant reoperation of the urethrostomy site in the remaining one case. The mean survival time was nine months. Among minor complications, recurrent cystitis was found. After the first surgery, all dogs retained full control over urination immediately after recovery from anesthesia. In one case that required reoperation, complete urinary incontinence occurred after the second procedure. The present findings suggest that hybrid surgery can be used to treat distal urethral tumors. Qualification for surgery must be limited to bitches with tumors of the distal urethra and without metastases, without the possibility of using other surgical methods, and with the owner’s full acceptance of the risk of complications.

## 1. Introduction

The most common form of canine urinary tract cancer is transitional cell carcinoma (TCC), accounting for up to 2% of all malignancies of this species [1]. In the literature, urethral tumors are mainly described as transitional epithelial tumors, most often affecting bitches [2,3,4].

Since extensive tumors of the distal urethra in female dogs rarely allow for complete removal and simultaneous reattachment of the severed urethra using an end-to-end technique, they may require such approaches as a prepubic urethrostomy, vaginourethroplasty, or conservative management [5,6]. The technical possibilities of vaginoplasty are limited, and prepubic urethrostomy should be treated only as a palliative form of treatment [5,6]. Among other palliative methods of treatment of patients with extensive tumors of the urethra we can differentiate cystostomy [7,8], urethral stenting [7,8,9], and transurethral resection [10].

Our team developed a novel hybrid surgical technique for the treatment of proliferative lesions of the distal urethra and presented its usage on the cadavers of female dogs [11]. We evaluated this method for radical lesion removal (with the theoretical possibility of maintaining a healthy tissue margin) while maintaining an undisturbed urine outflow.

The aim of this research was to evaluate the effectiveness and safety of this new surgical technique in the treatment of proliferative lesions of the distal urethra in cases requiring preservation of oncological margins. This study presents first retrospective results of our inquiry in three female dogs.

## 2. Materials and Methods

### 2.1. Patient Selection

**Figure 1:**
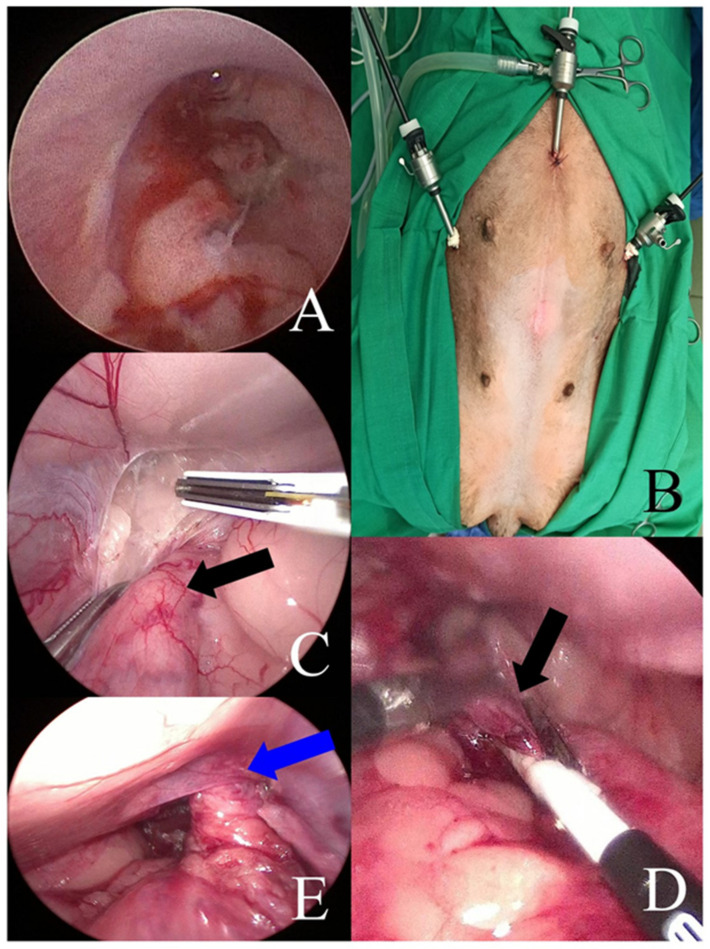
(**A**) Endoscopic image of transitional cell carcinoma tumor inside the lumen of the urethra. (**B**) Trocar system during hybrid surgery. (**C**) Laparoscopic intraoperative image of urethra dissection, black arrow—urethra. (**D**) Intraoperative endoscopic image of the urethra cut transversely (black arrow). (**E**) Intraoperative laparoscopic image of the urethrostomy site (blue arrow).

The inclusion criteria for this study were female dogs with distal urethral tumors, without metastatic lesions present. All operated female dogs were referred to the Department and Clinic of Surgery, Faculty of Veterinary Medicine, Wroclaw University of Environmental and Life Sciences with dysuria and hematuria. The procedures were performed by the same veterinary surgeon (PP). Standard aseptic techniques were followed for all three dogs during the hybrid surgical procedures. The study was retrospectively approved by the ethics committee of the Wroclaw University of Environmental and Life Sciences (Faculty of Veterinary Medicine Animal Welfare Advisory Team). All animal owners signed a consent form after explaining the details of anesthesia and hybrid surgery procedures and their associated risks. Complete blood cell count and biochemistry profiling were performed. All three patients underwent a clinical examination, including a rectal exam. To detect possible metastatic changes and assess the extent of the urethral tumor, computed tomography of the abdominal and thoracic cavities was performed. In addition, a urethrocystoscopy was performed, during which a fine-needle biopsy was taken under optical control Figure 1A. Female dogs qualified for surgery had changes limited to the urethra, without local and distant metastases. The excised tumors were secured for histopathological examination.

### 2.2. Anesthesia and Antibiotic Prophylaxis

Patients were premedicated intramuscularly with a mixture of dexmedetomidine (Dexdomitor, Orion Pharma, Wels, Austria) at a dose of 5 mcg/kg with methadone (Comfortan, Dechra, Bladel, Netherlands) at a dose of 0.2 mg/kg. General anesthesia was induced with propofol (initial dose 1 mg/kg to effect; Scanofol, ScanVet, Gniezno, Poland) and maintained with isoflurane (Isovet, Piramal Healthcare, Surrey, UK) in oxygen. Analgesia was provided by fentanyl bolus (2.5 mcg/kg) followed by constant rate infusion (0.2 mcg/kg/min; Fentadon, Dechra, Bladel, Netherlands) and epidural neuraxial block (lignocainum hydrochloricum WZF 2%, Polfa Warszawa, Poland). Intravenous fluids (crystalloids) were infused at the rate of 5 mL/kg/h during the whole anesthesia in all dogs. The IV bolus of fluid (crystalloid or colloid at 5–10 mL/kg or 3–5 mL/kg, respectively) was administered when hypotension occurred. Throughout the surgery, body temperature, hemodynamic, and respiratory parameters were monitored constantly. Postoperatively to reduce pain, the patients received buprenorphine (Bupaq Multidose, Orion Pharma, Wels, Austria) at a dose of 20 mcg/kg every 8 h for the next 3 days, and meloxicam (Metacam, Boehringer Ingelheim, Germany), also for 3 days—initially at a dose of 0.2 mg/kg, then 0.1 mg/kg. Additionally, on the day of the surgery, patients were given metamizole (Pyralgivet, Vet-Agro, Lublin, Poland) at a dose of 25–50 mg/kg every 8 h.

The operated dogs received an antibiotic subcutaneously for 7 days. The first injection of amoxicillin with clavulanic acid (Synergal Inj. (140 + 35) mg/mL ScanVet, Gniezno, Poland) at a dose of 12.5 mg/kg was administered just before the surgery and repeated every 24 h after the surgery.

### 2.3. Hybrid Surgical Procedures

The protocol for the hybrid surgical procedures followed previously described steps [11]. Resection of distal urethral tumors was carried out using the novel hybrid surgical method that was presented on cadavers by Prządka et al. [11]. Before the patient was placed on the operating table, residual urine was removed through a bladder catheter. The patients were placed in dorsal recumbency (Trendelenburg position), with the pelvic region placed on the edge of the operating table for the surgical preparation of the vulva and vagina during the procedure. The laparoscopic column was placed behind the caudal side of the patient and the operators stood on both sides of the operating table for proper intraoperative visualization. All endoscopic equipment used for the laparoscopic procedure involved a 5 mm 30° scope and was acquired from the same manufacturer (Karl Storz SE & Co. KG; Tuttlingen, Germany).

The laparoscopic procedure started with the insertion of three trocars of 5 mm in diameter. The first trocar, for laparoscopic optics, was inserted using the Hasson method at the level of the umbilicus [12]. After insufflation of the abdominal cavity (CO_2_ pressure of 8 mmHg), two consecutive trocars were placed caudal–laterally relative to the first optical trocar (Figure 1B).

**Figure 2:**
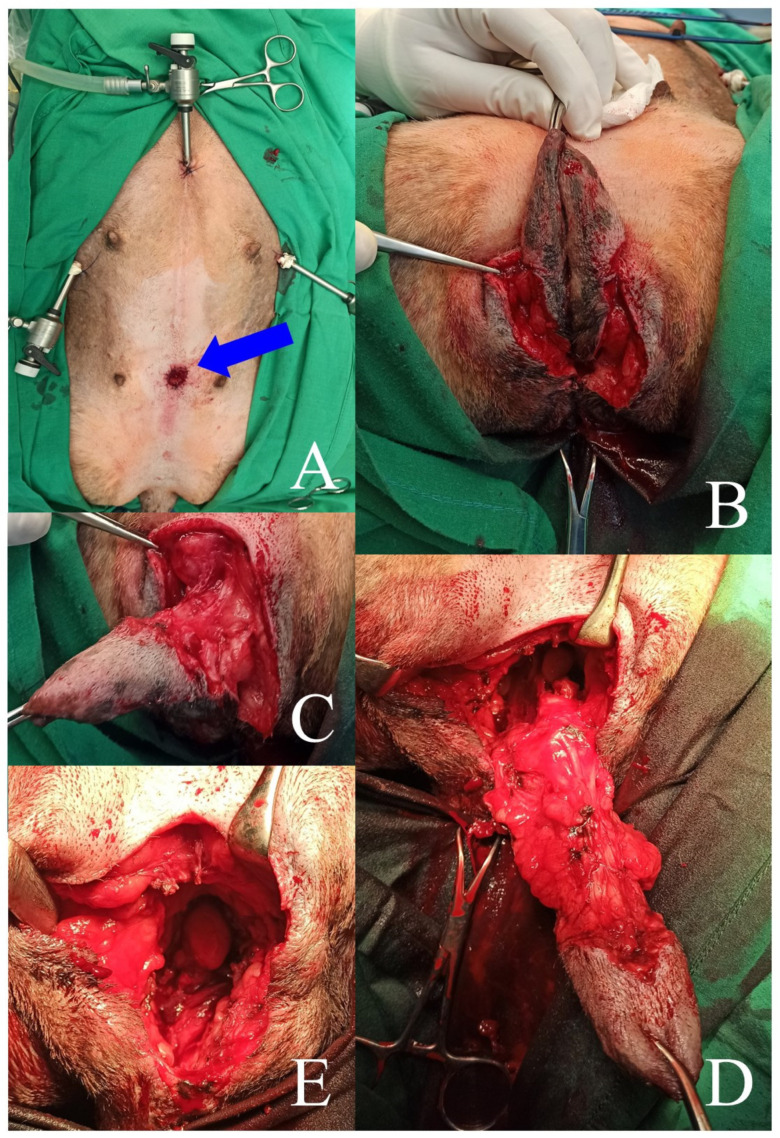
(**A**) Intraoperative image of medial prepubic urethrostomy—blue arrow. (**B**) Cutaneous incision line in vulvovaginectomy. (**C**,**D**) Preparation of the vulva and vaginal vestibule during hybrid surgery. (**E**) Intraoperative external image of the wound after vulva removal.

**Figure 3:**
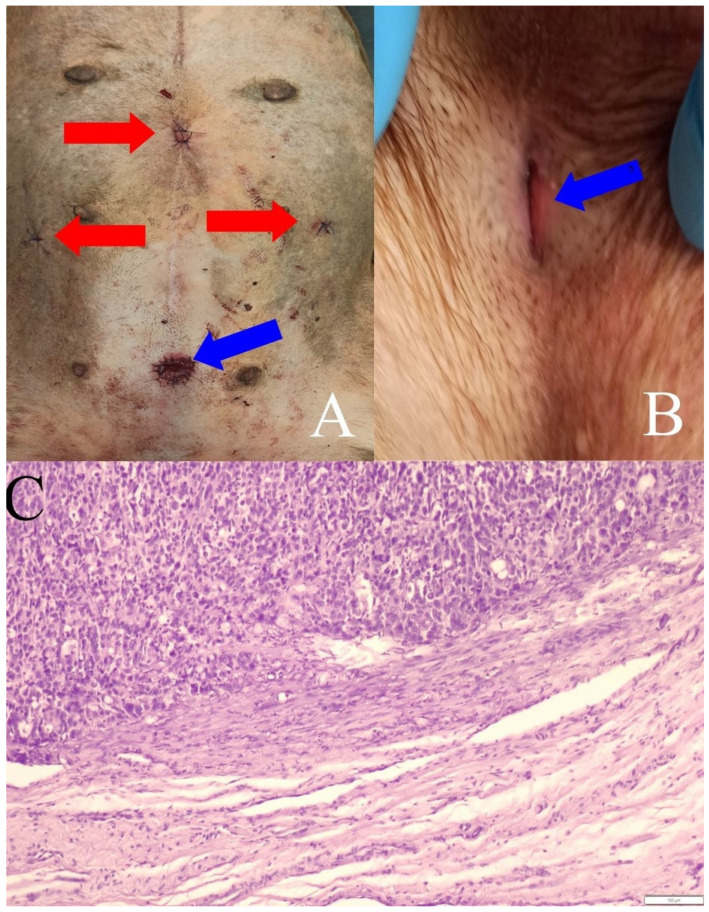
(**A**) Postoperative trocar wounds—red arrows, and prepubic urethrostomy wounds—blue arrow. (**B**) Prepubic urethrostomy image two months after surgery (blue arrow—urethrostomy). (**C**) Transitional carcinoma cells at the border of healthy connective tissue. HE staining, 100× (scale bar 100 µm).

Each time, the abdominal cavity was generally assessed. The procedure started with the dissection of the uterine stump with different types of vessel-sealing devices (BiCision, BiSect, Erbe Vio 3, Tübingen, Germany) used alternately. The operators used laparoscopic forceps to open and bluntly dissect the vesicogenital and pubovesical pouches, and subsequently, fatty tissue that surrounded the urethra, uterine stump, and vagina (Figure 1C). The vessels were closed with the above-mentioned sealing devices successively. First, the distal part of the urethra was dissected, after which the operator made a transverse cut with a vessel-sealing device (BiCision) just in front of the tumor, on the side of the bladder. This site was chosen subjectively, based on the macroscopic appearance in the laparoscopic image of the dissected urethra (significant widening of the affected part). The difference in the structure, assessed by its hardness, of the altered and unchanged sections of urethra was judged by squeezing it very gently with Maryland laparoscopic forceps, starting from the bladder. Before cutting the urethra, the residual urine was removed again from the bladder and the catheter was taken out. The urethra was cut transversely and its caudal part was laparoscopically dissected as far caudally as intraoperatively possible, along with the uterine stump (Figure 1D). After that, the operators stitched the proximal part of the urethra to the edges of the skin wound, which was made for this purpose with a scalpel blade No. 11, at a site located 2–3 cm cranially to the pubic symphysis. The wound measured 1–1.5 cm in length and penetrated through the entire thickness of the abdominal wall at the level of the linea alba. Under the control of the laparoscope, the proximal part of the urethra was grabbed with laparoscopic forceps, and then passed through the prepubic incision in the abdominal wall. The edges of the urethra were evened out with surgical scissors and then sewn to the skin margins with single interrupted non-absorbable material (Dafilon 4-0, B Braun, Rubi, Spain) (Figure 1E and 2A). The next stage involved the dissection of the vulva and distal part of the urethra and vagina (the part not previously dissected by laparoscopy) using open surgery. The technique proposed by Bilbrey et al. [13] was used to perform vulvovaginectomy. A fusiform incision of the skin was made around the vulva. The deeper tissues were sharply dissected from the labia and vestibule. The constrictor vestibule and constrictor vulvae muscles were dissected from the vestibule. The dorsal labial branches of the ventral perineal artery were coagulated. The dissection was performed cranially around the vagina by transecting the ischiocavernosus and ischiourethralis muscles and passing cranially between the paired levator ani muscles to the level of the cervix. The vaginal branches of the vaginal artery and vein, as well as the uterine arteries and their branches to the vagina, were coagulated (Figure 2B,C). This procedure allowed for the final dissection of the vulva and vagina, along with the rest of the urethra. Separation of these organs from the surrounding tissues allowed them to be removed from the abdominal cavity and pelvis through a surgical wound made at the level of the vulva (Figure 2D,E). The wound at the level of the perineum was closed in layers after the removal of the vulva and vagina, starting with a single purse-string suture inserted as deeply as possible into the pelvic lumen between the pelvic muscles and rectal wall. Subsequently, single interrupted sutures were placed on the muscles and subcutaneous tissue (Monosyn 0, B Braun, Rubi, Spain). The skin wound was sealed with single vertical mattress sutures. After performing open surgery and reobtaining pneumoperitoneum, the surgical field was assessed laparoscopically, and the trocars were removed. The trocar wounds were closed through all layers of the abdominal wall with single interrupted sutures of non-resorbable material (Dafilon 0, B Braun, Rubi, Spain) (Figure 3A,B).

### 2.4. Postoperative Care, Follow-Up, and Cyto-Histopathology Evaluation

On the second day after discharge, the patients were under the supervision of the referring veterinarian most of the time. The operated dogs had a follow-up visit to perform a physical examination and remove sutures only on the 10th day after the surgery. There were additional phone consultations with the owners of the dogs and their attending veterinarians before and after the control visit in cases that required it. The owners were questioned about any information regarding the health of their dogs, which may be the result of the surgical procedure as well as the previously diagnosed neoplastic disease. In the event of animal death, owners were also asked to inform the authors of the time and possible causes.

Cytological preparations, made from the biopsy material taken during the endoscopic examination, were stained with hematoxylin and eosin.

The resected fragments of the urethra as well as the fragments excised during the evening of the edges of the urethra were fixed in 10% formalin, and after obtaining histopathological preparations, they were also stained with hematoxylin and eosin.

The cytological and histopathological slides were observed under an Olympus BX53 microscope coupled with an Olympus UC90 camera (Olympus, Tokyo, Japan). For acquisition, the cellSens Standard V1 software was used (Olympus, Tokyo, Japan).

## 3. Results

### 3.1. Clinical Study

Three previously castrated female dogs underwent a hybrid surgical removal of the urethra because of neoplastic changes (French Bulldog, American Staffordshire Terrier, German Pointer) aged 9, 12, and 10 years, respectively, and with a body weight of 11, 24, and 31 kg, respectively.

Strenuous and painful urination was concluded from all three dogs’ medical history as well as their physical examination. A solid and hard tumor was palpated during the rectal examination at the level of the urethra. Urethrocystoscopy revealed a strongly narrowed urethral lumen, that prevented endoscopic examination of the bladder. Changes in the urethra ranged from the level of its orifice to the vestibule of the vagina. Simultaneous vaginoscopic examination showed no changes within the vaginal vestibule or the vagina itself.

Qualified dogs underwent contrast-enhanced computed tomography, which showed proliferative changes limited only to the urethra, with no metastatic changes in the abdominal cavity, pelvis, and thorax. The results of blood tests (CBC, biochemistry) were within normal limits and allowed for anesthesia and surgery.

The result of the cytological examination indicated a transitional cell carcinoma (TCC), which was confirmed by histopathological examination of the removed altered urethra. At the same time, in two of three cases conclusion from the histopathological examination of the resected urethra was that the margin of healthy tissue was preserved. (Figure 3C). The microscopic examination of the histopathological specimens from intraoperative specimens of the urethra tumor showed the weaving of a typical well-differentiated transitional cell carcinoma (carcinoma urothelial). In two female dogs, no neoplastic cells were observed in the connective tissue surrounding the urethral section in the incision line from the urethrostomy side.

In one case (French Bulldog), neoplastic cells were present in the incision line after urethrostomy and a decision was made to reoperate. During the second procedure, altered tissue fragments with a larger margin of healthy tissue were excised, which was confirmed by a second histopathological examination.

All three surgical procedures were possible to perform with the hybrid technique, without the need for conversion, i.e., transition to open surgery during the operation. All three patients were able to get up and move around without any problems immediately after waking up from the anesthesia. Control of urination was also quickly regained, with no signs of urinary incontinence. In one dog (French Bulldog), due to the need for reoperation after the assessment of the histopathological margin, the last procedure required resection of almost the entire urethra up to the anterior segment of the bladder cervix, which resulted in complete incontinence. This was accepted by the owner. All of the surgical wounds healed by primary intention (Figure 3B).

The mean survival time was 9 months (French Bulldog—8 months; American Staffordshire Terrier—13 months; German Pointer—6 months). At the same time, based only on the information obtained from the owners over the phone, we concluded that the cause of death was not related to the procedures performed or the diagnosed TCC of the urethra. None of the pets owners gave consent for a necropsy. As for the causes of death, they reported: a communication accident (French Bulldog), euthanasia because of intervertebral disc disease (IVDD) paralysis (American Staffordshire Terrier), and for the German Pointer the cause of death was unknown—the dog did not wake up one day in the morning.

### 3.2. Complications

Among the complications related to the procedure itself, one should mention recurrent cystitis, which was diagnosed by the primary care veterinarians of the first two operated dogs. Urinary tract inflammation was recognized twice in the American Staffordshire Terrier and four times in the French Bulldog. In the last patient, a margin of healthy tissue was not obtained during the first urethrostomy and an additional procedure was necessary, which resulted in permanent urinary incontinence.

## 4. Discussion

A novel hybrid surgical approach to neoplastic lesions in the distal part of the urethra was possible to conduct in all of the three female dogs, qualified for the operation. The fact that the conversion to open surgery was not necessary shows the potential advantages of the presented procedure. At the same time, the average survival time of 9 months allows us to conclude that the described procedure is not only feasible but also safe for the patient. 

According to the information obtained from the owners, in at least two cases, the cause of death of the female dogs operated on was neither surgery nor a tumor of the urethra. At the same time, no autopsy was performed in any of the cases, which made it impossible to accurately identify the causes of death.

Survival after surgery ranged from 6 to 13 months, without chemotherapy and other medications such as the anti-inflammatory piroxicam. It was the sole decision of the owners not to subject their pets to the added burden of taking medication on a daily basis, despite the clear implications.

No major complications such as hemorrhage or surgical site infection occurred in any of the operated dogs. The only recorded complication was the need for reoperation of the stoma to obtain a healthy tissue margin, which led to permanent urinary incontinence in one of the operated bitches.

Midline prepubic urethrostomy was performed in all operated female dogs, which is associated with a lower risk of urine leak onto the limb during voiding. Indications for urethrostomy in companion animals include the presence of neoplastic lesions or extensive injuries of the distal urinary tract [11,14,15]. Based on the authors’ scientific research [16] as well as according to the available literature [17,18], laparoscopic assistance is an ideal approach for performing prepubic urethrostomy. It is also superior to the same procedure performed by the classical method, where an extensive surgical wound lateral to the laparotomy incision is required to carry out the urethrostomy [16]. The open surgical approach may lead to complications such as leakage of urine on the limb, irritation and chronic dermatitis [14,16,17,19,20]. The method presented in the study allowed for the urethrostomy to be performed along the course of the linea alba, cranially to the symphysis of the pubis, minimizing the risk of the above-described complications. No such issues were reported by their main care veterinarians and the owners of the operated dogs through the whole observation period after the surgery.

One of the bitches developed complete urinary incontinence, but only after a second surgical intervention. Nevertheless, after the first procedures, all three operated animals maintained full control over micturition. This was probably due to the extent of the removed urethra, as the lowest risk of postoperative urinary incontinence was reported in the case of a urethral removal of one-third to one-half of its length measured from its distal part [5,11,13,21]. White et al. [5], using the above-mentioned indicators, conducted vaginourethroplasty for the treatment of urethral obstruction in six neutered female dogs, showing good long-term outcomes in most cases. The approach used in this study enabled radical removal of the target lesion and further assessments of the tumor recurrence, allowing timely treatment [5,11].

The designed hybrid procedure aimed to enable the removal of the tumor in the distal part of the urethra and to perform prepubic urethrostomy. Different surgical approaches to pelvic organs include access via the caudal abdomen, sagittal pelvic osteotomy, and a bilateral pubic and ischial osteotomy [15,22,23,24]. The caudal abdomen access appears to be less invasive than the other options. Pelvic osteotomy is great for the surgeon because it improves intraoperative access and visibility; however, compared with caudal abdomen access it significantly increases the extent of trauma [16]. In our opinion, both open surgery and our hybrid technique allow access and control of the operating field. However, the hybrid method involves less trauma, which is the result of a minimally invasive approach [16].

Endoscopic surgery has many advantages over open surgery for certain types of procedures, as previous studies have shown [16,25,26]. The hybrid technique in the opinion of the authors is less invasive than the same procedure performed with the open technique. In addition, a compelling solution may be the intraoperative use of cystoscopy to determine the boundaries of the changed urethra before its resection. However, this requires an appropriate width of the lumen of the cancerous urethra, enabling the cystoscope to be inserted into the bladder. The cases described in the article did not allow for a cystoscopic examination to determine the borders of the tumor because the lumen of the urethra was too narrow.

One of the major complications that may occur during urogenital surgery is ureteral injuries [27,28,29,30]. No case of ureteral wall damage was observed in the present study; however, based on the scientific reports, special care should be taken to prevent this type of damage during hybrid surgery [11,16].

Other postoperative complications after the presented surgical technique include recurrent urinary tract infection, which is probably the result of the performed urethrostomy, which increases the risk of infection. Bladder infection has also been found in operated dogs by primary care veterinarians, which, in the authors’ opinion, should be the reason for continuous monitoring of all patients with urethrostomy in order to quickly diagnose possible cystitis. 

The main limitation of this study was the small number of cases, however, tumors of the urinary tract account for <2% of all tumors seen in dogs. The rigorous surgery qualification process, concerning the localization of the tumor (restricted only to the urethra) and the absence of metastatic lesions considerably lower the number of cases, even in reference centers. A significant limitation of the presented surgical technique is the lack of wide accessibility to laparoscopy in veterinary medicine all over the world, which means that this procedure may not be performed anywhere, despite indications. According to the authors’ knowledge, based on the previous study [16], the most demanding stage of the procedure is the dissection of the pelvic part of the urethra. A lack of appropriate laparoscopic skills may be problematic for the surgeons performing only basic surgical procedures endoscopically. An additional difficulty is the retrospective evaluation of the presented cases and the inability to monitor the patients daily during postoperative follow-up. The authors of this study are aware of the limitations of the objective evaluation of the presented surgical technique, based only on three cases, requiring further research.

## 5. Conclusions

The results obtained on a very small group of operated animals allow for a conclusion that the technique presented by the authors earlier on dog cadavers is safe to use and feasible on live patients. The advanced age of the operated bitches and the probable presence of comorbidities do not allow for an unequivocal long-term assessment of survival time after choosing the described method of treatment. In our opinion, patients for treatment with the hybrid surgical method should be rigorously selected in the absence of another, less invasive method of treatment and with full acceptance by the owners of possible postoperative complications, such as urinary incontinence or cancer recurrence.

## Data Availability

The data generated in this study are in this article.

## References

[1] Malfassi L., Fidanzio F., Sala M., Marcarini S., Mazza G., Carrara N., Pavesi S., Gnudi G., Urso G., Dolera M. (2021). A Combined Protocol with Piroxicam, Chemotherapy, and Whole Pelvic Irradiation with Simultaneous Boost Volumetric Modulated Arc Radiotherapy for Muscle-Invasive Canine Urinary Transitional Cell Carcinoma: First Clinical Experience. J. Vet. Med. Sci..

[2] Priester W.A., McKay F.W. (1980). The Occurrence of Tumors in Domestic Animals. Natl. Cancer Inst. Monogr..

[3] Norris A.M., Laing E.J., Valli V.E., Withrow S.J., Macy D.W., Ogilvie G.K., Tomlinson J., McCaw D., Pidgeon G., Jacobs R.M. (1992). Canine Bladder and Urethral Tumors: A Retrospective Study of 115 Cases (1980–1985). J. Vet. Intern. Med..

[4] Knapp D.W., Glickman N.W., Denicola D.B., Bonney P.L., Lin T.L., Glickman L.T. (2000). Naturally-Occurring Canine Transitional Cell Carcinoma of the Urinary Bladder A Relevant Model of Human Invasive Bladder Cancer. Urol. Oncol..

[5] White R.N., Davies J.V., Gregory S.P. (1996). Vaginourethroplasty for Treatment of Urethral Obstruction in the Bitch. Vet. Surg..

[6] Davies J.V., Read H.M. (1990). Sagittal Pubic Osteotomy in the Investigation and Treatment of Intrapelvic Neoplasia in the Dog. J. Small Anim. Pract..

[7] Weisse C., Berent A., Todd K., Clifford C., Solomon J. (2006). Evaluation of Palliative Stenting for Management of Malignant Urethral Obstructions in Dogs. J. Am. Vet. Med. Assoc..

[8] McMillan S.K., Knapp D.W., Ramos-Vara J.A., Bonney P.L., Adams L.G. (2012). Outcome of Urethral Stent Placement for Management of Urethral Obstruction Secondary to Transitional Cell Carcinoma in Dogs: 19 Cases (2007–2010). J. Am. Vet. Med. Assoc..

[9] Blackburn A.L., Berent A.C., Weisse C.W., Brown D.C. (2013). Evaluation of Outcome Following Urethral Stent Placement for the Treatment of Obstructive Carcinoma of the Urethra in Dogs: 42 Cases (2004–2008). J. Am. Vet. Med. Assoc..

[10] Liptak J.M., Brutscher S.P., Monnet E., Dernell W.S., Twedt D.C., Kazmierski K.J., Walter C.U., Mullins M.N., Larue S.M., Withrow S.J. (2004). Transurethral Resection in the Management of Urethral and Prostatic Neoplasia in 6 Dogs. Vet. Surg..

[11] Prządka P., Liszka B., Antończyk A., Gąsior L., Kiełbowicz Z. (2022). Novel Surgical Approach to Neoplastic Lesions in the Distal Part of the Urethra: A Pilot Cadaver Study Comparing Open and Hybrid Techniques. Vet. Comp. Oncol..

[12] Hasson H.M. (1971). A Modified Instrument and Method for Laparoscopy. Am. J. Obstet. Gynecol..

[13] Bilbrey S.A., Withrow S.J., Klein M.K., Bennett R.A., Norris A.M., Gofton N., DeHoff W. (1989). Vulvovaginectomy and Perineal Urethrostomy for Neoplasms of the Vulva and Vagina. Vet. Surg..

[14] Risselada M., Rooster H., Waelbers T., Geffen C., Vermote K., Kramer M. (2006). A Prepubic Urethrostomy in a Bitch after Resection of the Vagina and the Distal Part of the Urethra. Vlaams Diergeneeskd. Tijdschr..

[15] Salomon J.F., Deneuche A., Viguier E. (2004). Vaginectomy and Urethroplasty as a Treatment for Non-Pedunculated Vaginal Tumours in Four Bitches. J. Small Anim. Pract..

[16] Prządka P., Liszka B., Lachowska S., Dzimira S., Ciaputa R., Tunikowska J., Juźwiak Ł., Kucharski P., Rudno-Rudzińska J., Kiełbowicz Z. (2021). Case Report Laparoscopy-Assisted Pre-Pubic Urethrostomy as a Palliative Procedure for Resection of Distal Urethral Tumor in a Female Dog. BMC Vet. Res..

[17] Queiroga L.B., Lopes L.M.A., Gianotti G.C., Scherer S., Alievi M.M., de Castro Beck C.A. (2017). Laparoscopic-Assisted Prepubic Urethrostomy: Experimental Model in Rabbit. Cienc. Rural.

[18] Pinto Filho S.T.L., Oliveira M.T., Souza F.W., Dalmolin F., Hartmann H., Coutinho Júnior A.S., Schuster L.A.H., Beck C.A.C., Santos F.R.B., Feranti J.P.S. (2014). Laparoscopic-assisted prepubic urethrostomy in a cat with urethral stenosis. Semin. Ciências Agrárias.

[19] Baines S.J., Rennie S., White R.S. (2001). Prepubic Urethrostomy: A Long-Term Study in 16 Cats. Vet. Surg..

[20] Lipscomb V. (2004). Surgery of the Lower Urinary Tract in Dogs: 2. Urethral Surgery. Practice.

[21] Chambers J.T., Schwartz P.E. (1987). Mobilization of Anterior Vaginal Wall and Creation of a Neourethral Meatus for Vulvectomies Requiring Resection of the Distal Part of the Urethra. Surg. Gynecol. Obstet..

[22] Tarvin G., Patnaik A., Greene R. (1978). Primary Urethral Tumors in Dogs. J. Am. Vet. Med. Assoc..

[23] Allen S.W., Crowell W.A. (1991). Ventral Approach to the Pelvic Canal in the Female Dog. Vet. Surg..

[24] Muir P., Bjorling D.E. (1994). Ventral Approach to the Pelvic Canal in Two Dogs. Vet. Rec..

[25] Gower S., Mayhew P. (2008). Canine Laparoscopic and Laparoscopic-Assisted Ovariohysterectomy and Ovariectomy. Compend. Contin. Educ. Vet..

[26] Lee J.Y., Kim M.C. (2014). Comparison of Oxidative Stress Status in Dogs Undergoing Laparoscopic and Open Ovariectomy. J. Vet. Med. Sci..

[27] Dorairajan G., Rani P.R., Habeebullah S., Dorairajan L.N. (2004). Urological Injuries during Hysterectomies: A 6-Year Review. J. Obstet. Gynaecol. Res..

[28] Van Goethem B., Schaefers-Okkens A., Kirpensteijn J. (2006). Making a Rational Choice between Ovariectomy and Ovariohysterectomy in the Dog: A Discussion of the Benefits of Either Technique. Vet. Surg..

[29] Burks F.N., Santucci R.A. (2014). Management of Iatrogenic Ureteral Injury. Ther. Adv. Urol..

[30] Plater B.L., Lipscomb V.J. (2020). Treatment and Outcomes of Ureter Injuries Due to Ovariohysterectomy Complications in Cats and Dogs. J. Small Anim. Pract..

